# *PLOS Medicine* 2016 Reviewer and Editorial Board Thank You

**DOI:** 10.1371/journal.pmed.1002281

**Published:** 2017-03-20

PLOS and the *PLOS Medicine* team would like to express our sincere gratitude to everyone who lent us their expertise as an academic editor, as a guest editor or as a peer reviewer in 2016. We are fully dependent on the voluntary efforts of these experts to publish the journal for the academic community and the wider readership concerned with human health. Over the past year, *PLOS Medicine* relied on 116 academic and guest editors to manage the 1,070 articles submitted to the journal, which were assessed by more than 800 reviewers. Their efforts enabled the publication of more than 250 rigorous, Open Access papers.

The names of our 2016 editors that handled submitted manuscripts appear in the Supporting Information as S1 Editor List and as S1 Guest Editor List. Our reviewers appear in the Supporting Information as S1 Reviewer List. We are grateful for their dedication, generosity, and support of Open Access science. Thank you all.

## Supporting information

S1 Editor List Alphabetical list of 2016 *PLOS Medicine* Editorial Board Members. (PDF)

S1 Guest Editor List Alphabetical list of 2016 *PLOS Medicine* Guest Editors. (PDF)

S1 Reviewer List Alphabetical list of 2016 *PLOS Medicine* peer reviewers. (PDF)

## References

[1] (2016) *PLOS Medicine* 2015 Reviewer Thank You. PLoS Med 13(2): e1001983 doi: 10.1371/journal.pmed.1001983

[2] (2015) *PLOS Medicine* 2014 Reviewer Thank You. PLoS Med 12(2): e1001806 doi: 10.1371/journal.pmed.1001806

