# Supplementary material for: PLOS Medicine 2016 Reviewer and Editorial Board Thank You
Source: PLoS Med. 2017 Mar 20;14(3):e1002281. doi: 10.1371/journal.pmed.1002281 (PMC5358730; doi:10.1371/journal.pmed.1002281)
Supplement: S1 Editor List — (PDF) [file pmed.1002281.s001.pdf]

PLOS | MEDICINE

PLOS Medicine would like to thank all those who served on the Editorial Board in 2016:

Hans-Olov Adami
Marcus Altfeld
Hilda Bastian
Sanjay Basu
Andrew H. Beck
Zulfiqar A. Bhutta
Agnes Binagwaho
Carol Brayne
Karim Brohi
Peter Byass
Suzanne C. Cannegieter
Lucy C. Chappell
Matthias Egger
Eduardo L. Franco
Sam Gandy
Rebecca Freeman Grais
Wayne D. Hall
Stephan Harbarth
Phillipa J. Hay
Tom W.J. Huizinga
Lars Hviid
John P.A. Ioannidis
Rachel Jewkes
Aaron S. Kesselheim
Mirjam E.E. Kretzschmar
Sanjeev Krishna
Margaret E. Kruk
Claudia Langenberg
Tze Kin Lau
Richard Lehman
Cathryn Lewis
Marc Lipsitch
Nicola Low
Malcolm R. Macleod
Clara Menendez
John Z. Metcalfe
Philippa Middleton
Cosetta Minelli
Lynne Meryl Mofenson
Megan Murray
Jenny E. Myers
Marie-Louise Newell
Olivier Neyrolles
Abdisalan Mohamed Noor
David Osrin
Madhukar Pai
Anushka Patel
Andrew Prentice
Kazem Rahimi
Donald A. Redelmeier
Andrew S.C Rice
Stephen John Rogerson
Stephanie L. Sansom
Mathuram Santosham
Barbara L. Shacklett
Steven D. Shapiro
Aziz Sheikh
Nandi Siegfried
Amit Singal
Mervyn Singer
Thomas A. Smith
Maarten W. Taal
Mark Tomlinson
Alexander C. Tsai
James K. Tumwine
Jean-Louis Vincent
Lorenz von Seidlein
Sheri D. Weiser
Clifford J. Woolf
