# Supplementary material for: PLOS Medicine 2016 Reviewer and Editorial Board Thank You
Source: PLoS Med. 2017 Mar 20;14(3):e1002281. doi: 10.1371/journal.pmed.1002281 (PMC5358730; doi:10.1371/journal.pmed.1002281)
Supplement: S1 Guest Editor List — (PDF) [file pmed.1002281.s002.pdf]

PLOS Medicine would like to thank all those who served as Guest Academic Editors in 2016:

Tony Blakely
Tom Boyles
Clarissa Brocklehurst
Druin Burch
Carlos Caldas
Mike Clarke
Silvia de Sanjosé
Luis Diaz
Benjamin Djulbegovic
Nathan Ford
Adriane Fugh-Berman
Paul Garner
Ruth Gilbert
David Henry
William Herman
Pekka Kannus
Katharina Kranzer
Marc Ladanyi
Justin Lessler
Joel Lexchin
Stephen Luby
Ronald Ma
Elaine R. Mardis
Florian Markowetz
Martin McKee
Bruce L. Miller
Edward Mills
John Minna
Steven Moore
Ivo Mueller
Paolo Muraro
Thomas Novotny
Jonathan Rees
Jurgen Rehm
Giuseppe Remuzzi
Steven Riley
Sydney Rosen
Martin Schreiber
Paul Shekelle
Lone Simonsen
Andreas Stuck
Amitabh Suthar
Matthew Todd
Cecile Viboud
Timothy Vyse
Nicholas Wareham
Martin White
Steven Woloshin
