# Supplementary material for: PLOS Medicine 2016 Reviewer and Editorial Board Thank You
Source: PLoS Med. 2017 Mar 20;14(3):e1002281. doi: 10.1371/journal.pmed.1002281 (PMC5358730; doi:10.1371/journal.pmed.1002281)
Supplement: S1 Reviewer List — (PDF) [file pmed.1002281.s003.pdf]

PLOS Medicine would like to thank all those who reviewed on behalf of the journal in 2016:

Rasmus á Rogvi
Ali Abbasi
Tarekegn Abeku
Bibhav Acharya
Adine Adonis
Y. Agrawal
Hyeong Sik Ahn
Zarko Alfirevic
Meredith Alger
Hamid Ali
Ash Alizadeh
Marcella Alsan
Marjan Alssema
Craig Anderson
Karen Anderson
Oskar Angerås
Xavier Anglaret
Jeannie Annan
Kaarin Anstey
John Aponte
Alberto Ascherio
Phil Asherson
Lauren Aubert
Nathalie Auger
Mauricio Avendano
Dimitrios Avramopoulos
Andrew Azman
Rajiv Bahl
Patricia Bailey
Janis Baird
Suzanne Baker
David R Baldwin
Jangu Banatvala
Amitava Banerjee
Krishna Baradhi
Ruben Barakat
Corrado Barbui
Deborah Barnes
Scott Barnhart
Till Bärnighausen
David Batty
Daniel Baumhoer
Scott Beattie
Jasper Been
James Beeson
Lazaros Belbasis
Phillip Bennett
Anick Bérard
Rene Bernards
Brookie Best
Arvin Bhana
Prashant Bharadwaj
Achuyt Bhattarai
Kirsten Bibbins-Domingo
Alessandro Biffi
Charles Billington
Sean Blackwell
Tony Blakely
John Blevins
Meridith Blevins
Michael Bloch
Gladys Block
Hélène Blons
Barry Bloom
Theodora Bloom
Elizabeth Blue
Annick Bogaerts
Maureen Bolon
Marialaura Bonaccio
Maciej Boni
Hervé Bonnefoi
Marc Bonnefoy
Heather Boonstra
Erika Borella
Soo Borson
Xavier Bosch
Andrea Bosman
Dimitrios Boumpas
Malaz Bousanti
Paul Boutros
Andrée-Anne Bouvette-Turcot
Pierre Bouzat
Philip Boyce
Tom Boyles
Paul Brand
Jose Bras
Michael Bretthauer
Marc Brisson
Allen Brown
Colin Brown

Peter J. Brown
Adam Brufsky
Germaine Buck Louis
Christophe Bula
Peter Bull
Stephen Burgess
James F Burke
Louis Buscail
Helen Bygrave
George Calin
Txema Calleja
Dominique Campion
Charles Cantor
Simon Capewell
Marly Cardoso
Daniel Carlat
David Carr
Isabelle Carriere
Richard Caselli
Adithya Cattamanchi
Simon Cauchemez
Uta Ceglarek
Brian Chan
Doris Sau Man Chan
Juliana Chan
Timothy Chan
Chin-Kuo Chang
Larry Chang
Nilanjan Chatterjee
Ian Cheeseman
Yih-Sharng Chen
Zhengming Chen
Rose Cheney
Alice Chen-Plotkin
Andrea Ciaranello
Olga Ciccarelli
Iza Ciglenecki
Andrea Cipriani
Jordi Clarimon
Jordi Clarimón
Cari Clark
Jack Clark
Sam Clark
Mike Clarke
Deborah Clegg
Christine Cleghorn
Carl Cohen
Craig Cohen
Myron Cohen
Ted Cohen
Tim Cole
Simon Collin
Sabine Colnot
Miriam Colombo
Alexis Conason
Francesca Conradie
Nancy Cook
E. Copson
Tony Cornford
Carol Coupland
Philip Coyne
Alister Craig
John Crawford
Peter Croft
Liwang Cui
C. Munro Cullum
Ruth Cunningham
Scott Curry
Dana Dabelea
Stephanie Dancer
Brian Dannemann
Jean-François Dartigues
Jai Das
Neil Davies
Daniel Davis
Ank de Jonge
Harry de Koning
Agnes Dechartres
Jacqueline Deen
Alessandra Dei Cas
Christian Delles
Kevin Delucchi
Nikos Demiris
Fiona Denison
Michael Denkinger
Delan Devakumar
Michael Dewey
Juan Dewez
Kumar Dharmarajan
Haryana Dhillon
Luis Diaz
Mireia Diaz Sanchis
Xavier Ding
Emilio Dirlikov
Roger Dixon
Hiroko Dodge
Arjen Dondorp
Christl Donnelly
Saskie Dorman
Peter Doshi
David Dowdy
Alison Drake

Charles Drake
John Drake
Elizabeth Draper
Shufa Du
Philipp du Cros
Radboud Duintjer Tebbens
Adriano Duse
Jeffrey Eaton
Philip Eckhoff
Michael Eikmans
Mohamed Eladawy
Matthew Ellis
Holger Eltzschig
Jonathan Emberson
Sean Emery
Ingunn Engebretsen
Mary-Anne Enoch
Jose Esparza
James Faghmous
Dennis Falzon
Xiujun Fan
David Fardo
Maha Farhat
Andrew Farmer
Cindy Farquhar
Mina Fazel
Seena Fazel
Jane Ferrie
Tobias Feuchtinger
Eric Fevre
Donna Fick
Sarah Fidler
Jose Figueroa
David Fisman
Stefan Flasche
Paul Fleming
Corinne Fligner
Peter Flom
Jose Florez
Robin Foà
Kwun Fong
Arnaud Fontanet
Nathan Ford
Nita Forouhi
Edward Fottrell
Guillaume Fournié
Chris Fox
Gregory Fox
Matthew Fox
Molly Franke
Timothy Frayling
Nick Freemantle
Theresa Freeman-Wang
Jonas Frisén
Jingyuan Fu
John Furler
Meghan Gabriel
Joshua Gagne
Edwin Gale
Alison Galvani
Mary Ganguli
Dingcheng Gao
Christopher Gardner
Paul Garner
Karen Gelmon
Emmanuelle Génin
Effrossyni Gkrania-Klotsas
Stanton Glantz
Richard Glassock
Julie Glavind
Vincent Gnanapragasam
Ajay Goel
Lee Gan Goh
Elizabeth Goldberg
Brett Gordon
Steven Gore
Roland Gosling
Patricia Graves
Laura Gray
Ronald Gray
Bryan Greenhouse
Nicola Greenlaw
Ed Gregg
Jamie Griffin
Sandeep Grover
Marica Grskovic
Elin Grundberg
Yi Guan
Li Guangwei
Rita Guerreiro
David Gunnell
Emily Gurley
Jessica Haberer
Judith Hahn
Nancy Haigwood
Damen Haile Mariam
Pranabashis Haldar
Prudence Hamade
Amresh Hanchate
Anandwardhan Hardikar
John Hardy
Olivier Harismendy

Bernard Harlow
Diane Harper
Jason Harris
Steve Harris
Ken Harvey
Ian Hastings
Mark Hatherill
Karin Hatzold
Phillipa J. Hay
Trey Hedden
Mary Hediger
Amanda Herbert
Matthew Herder
Karl Herholz
Peter Heutink
Sarah Hilmer
Swapnil Hiremath
Martin Hirsch
Jane Hirst
Katherine Hoadley
Leanne Hodson
Michael Hoffer
Daniel Hogan
Lars Holmberg
Henne Holstege
Jaco Homsy
Mehran Hosseini
Bo Hu
Maite Huarte
Jean Humphrey
Paul Hunter
Samia Hurst
Andrew Hutchings
Emily Hyle
Mohammad Ikram
Stamatina Iliodromiti
Collins Iwuji
Allison Jackson
James Jackson
Anders Jacobsen Skanderup
Karen Jacobson
Halliday Jane
A. Cecile Janssens
Jonathan Jay
Kurt Jellinger
Claire Jenkins
Jorgen Jensen
Irene Jillson
Mark Jit
Boo Johansson
Laura Johnson
Leigh Johnson
Edward Johnstone
Kate Jolly
Theodore Joyce
Naofumi Kagara
James Kahn
Helen Kales
Lisa Kalisch Ellett
Joan Kalyango
Satoshi Kaneko
Niranjan Kanesa-Thasan
Harin Karunajeewa
Martijn Katan
Michael Kattan
David Katzenstein
Alexander Kekulé
Sean Kennelly
George Kephart
Lars Kessing
Kamlesh Khunti
Kiran Khush
Monique Kilkenny
Peter Kilmarx
Catherine Kim
Hee-Jin Kim
Yohannes Kinfu
Nicholas King
Edward Kingdon
Carl Kirkwood
Spyros Kitsiou
Jeffrey Klausner
Eili Klein
Eric Klein
Paul Knight
Kristen Knutson
Kevin Kobylinski
Tracey Koehlmoos
Serena Koenig
Jan Koetsenruijter
Ann Kolanowski
Pete Kolsky
Udo Kontny
Olivier Koole
Frederick Korley
Cindy Körner
Andreas Kotsadam
Roger Kouyos
Alain Koyama
Katharina Kranzer
Andrea Kriska
Adam Kucharski

Anne Kuhlmann
Rohit Kulkarni
Janice Kwon
Carl Lachat
Louise Lafortune
Martin Lajous
Alastair Lamb
Claudio Lanata
Simon Langdon
David Larsen
Eric Larson
Estrella Lasry-Levy
Yocheved Laufer
Pirkka-Pekka Laurila
James Lavery
David Le Couteur
Kirsty Le Doare
David Leaf
Valerie Lebleu
David Lee
James Lee
Seung Heon Lee
Richard Lehman
Brian Lehmann
Jochen Lennerz
Philippe Lepage
Hannah Leslie
Justin Lessler
Guillaume Lettre
Daniel Leung
Michael Levin
Robert Levis
Joel Lexchin
Lanjuan Li
Min Li
Qifu Li
Benjamin Linas
Richard Lindley
Katherine Littler
Juan Llompart-Pou
Adam Locke
Christina Lockwood
William Lockwood
Stacy Loeb
Giancarlo Logroscino
Sherene Loi
Jamie Love
Marian Loveday
Julie A. Lovegrove
Sheng-Nan Lu
Gregory Lucas
Sebastian Lucas
Paul Lucassen
William Lydiatt
John Lynch
Valeriya Lyssenko
Ronald Ma
Freya Macmillan
Kristine Madsen
Luca Malcovati
Thomas Malek
Mats Målqvist
Justin Mandala
Ulrich Mansmann
Christophe Mariat
Paul Marik
Celina Martelli
Natasha Martin
Brian Martinson
Malek Massad
Colin Mathers
Fiona Matthews
Lynn Matthews
Soeren Mattke
Joan Maurel
Margaret May
Richard Mayeux
Mervyn Maze
Luca Mazzarella
David McAllister
Elizabeth McCarthy
James McCarthy
Catherine McCarty
Alex McConnachie
Johanna McEntyre
James McKerrow
Emma McMahon
John McNeil
Andrew McQuillin
Emma Meader
Nicholas Medland
S. Mehtar
Karina Meijer
Duane Mellor
Dick Menzies
Boyd Metzger
Florence Mgawadere
Christel Middeldorp
Michelle Mielke
Elizabeth Miller
Louis Miller
Pjm Milligan

Barbara Mintzes
Olivo Miotto
David Mischoulon
Friedrich Mittermayer
Susan Molchan
Yasmina Molero
Zoe Moodie
Andrew Moore
Kathleen Moore
Kerryn Moore
Kevin Moore
Siobhan Mor
Andrew Moran
Arden Morris
J. Glenn Morris Jr.
Amy Morrison
James Mortimer
Susan Morton
Steven Moss
Enrico Mossello
Louis Muglia
Simon Muhumuza
Luke Mullany
Marcus Munafo
Helen Murphy
Therese Murphy
Andrea Murru
Giovanni Musso
Monde Muyoyeta
Jenny Myers
Arnstein Mykletun
Paul Myles
Oliver Mytton
Steven Narod
Elena Naumova
Bruce Neal
Robert Nelson
Marion Nestle
Peter Neumann
Paul Newcombe
Louise Newman
Christelle Nguyen
Stephen Nicholls
Daan Nieboer
Jacek Niedziela
Christopher Nielsen
Katharina Nimptsch
Marloes Nitert
Peter Nordström
Jane Norman
Susan Norris
Bohdan Nosyk
Pierre Nouvellet
Myaing Nyunt
Max O'Donnell
Lucy Okell
Kelli O'Laughlin
Olushayo Olu
Chiadikaobi Onyike
Walter Orenstein
Lori Orlando
Matthew Page
Frederick Palm
Jenna Panter
Nickolas Papadopoulos
Elli Papaemmanuil
Pau Pastor
Jig Patel
Nim Pathy
Chris Pattillo
Chris Paul
Mical Paul
Daniel Payne
Jon Pedersen
Emmanuel Peprah
Rafael Perez-Escamilla
Leigh Perreault
Lars Åke Persson
Ruth Peters
Maya Petersen
Mark Petticrew
Audrey Pettifor
Ruth Pfeiffer
Curtis Pickering
Vincent Piguet
Kevin Pile
Didier Pittet
Virginia Pitzer
Richard Platt
Elizabeth Plimack
Katerina Politi
Jennifer Poti
Melanie Price
Martin Prince
Paul Pronyk
Chengxuan Qiu
Regina Rabinovich
Rosa Rademakers
Pratima Raghunathan
Md. Mizanur Rahman
Anita Raj
Sreeram Ramagopalan

Michael Ramharter
Didier Raoult
Ruwan Ratnayake
Fahad Razak
Karen Reckamp
Jurgen Rehm
Nicholas Reich
Simone Reppermund
Blanca Restrepo
Charles Reynolds
J. Brent Richards
Marty Richardson
Annette Rid
Steffi Riedel-Heller
Steven Riley
Eileen Rillamas-Sun
Giovanni Ristori
Emmert Roberts
Deirdre Robertson
Leanne Robinson
Timothy Robinson
Peter Rockers
Michael Roerecke
Joan Rogers
Pejman Rohani
Tina Rönn
Monique Roobol
Adam Rose
Rafael Rosell
Philip Rosenthal
Lionel Rostaing
Mary Jane Rotheram-Borus
Michael Rubin
Igor Rudan
Tom Russ
Michael Rutter
Kerry-Anne Rye
David Salisbury
Louis-Rachid Salmi
Jeffrey Samet
Manj Sandhu
Daisuke Sano
Stephanie Sarantopoulos
Ponnusamy Saravanan
Franco Sassi
Naveed Sattar
Peter Scarborough
Hans Scherer
Gordon Schiff
Heather Schofield
Julia Schofield
C. Mary Schooling
Heribert Schunkert
Laura Schwab-Reese
Pippa Scott
Kim Thuy Seelinger
Nicholas Selby
Holly Shakya
Idan Shalev
Aditya Sharma
Paul Shekelle
Sasha Shepperd
Catherine Sherwin
Priya Shete
Jeremy Shiffman
Mark Siedner
John Sievenpiper
Rebecca Simmons
Gregory Simon
Richard Sinert
Valérie Siroux
Kristel Sleegers
Elizabeth Smith
Robert Snow
Jonathan Snowden
Alina Solomon
Regan Solomons
Dahye Song
Maria Pia Sormani
Michael Spagat
Sandra Springer
Nicholas Stacey
Kimber Stanhope
Charles Stanley
Margaret Stanley
Sergio Starkstein
John Starr
Pär Stattin
Aryeh Stein
Dov Stekel
Barney Stephenson
Peter Sterk
Robert Stewart
Ewout Steyerberg
Eileen Stillwaggon
Hanni Stoklosa
Nathalie Streichenberger
Dan Strickman
Jeffrey Stringer
Erich Sturgis
Ramnath Subbaraman
Omar Sued

Karsten Suhre
Mark Sullivan
Dipika Sur
Jeremy Sussman
Amitabh Suthar
Charles Swanton
Daniel Swerdlow
Cameron Swift
Elizabeth Swisher
Matthew Sydes
Harry Tagbor
Shahrad Taheri
Lindsey Smith Taillie
Hajime Takechi
Wei Tang
Edmond Teng
Nikesh Thiruchelvam
Harsha Thirumurthy
Brett Thombs
John Thompson
Rebecca Thornton
Kamala Thriemer
Guy Thwaites
Jeanne Tie
Mary Tierney
Nicholas Timpson
Laurie Tomlinson
Marialena Trivella
Alexandra Trkola
Alexander Tsai
Katsuya Tsuchihara
Janet Turan
Venkatachalam Udhayakumar
Daniel Umpierre
Frederick Unverzagt
Alexander Upfill-Brown
Olalekan Uthman
Matti Uusitupa
Alex Valadka
Alfonso Valencia
Michael Valenzuela
Nicola Valeri
Wim Van Biesen
Reinout van Crevel
Floris Van Dam
Cornelis van de Velde
Janneke van de Wijgert
Rafael Van den Bergh
Felix Van der Meer
Philip Van der Wees
Sean van Diepen
Jean-Pierre van Geertruyden
Michel van Herp
Marinus Van IJzendoorn
Anne Van Kempen
Hanneke van Laarhoven
Pieter Van Vlierberghe
Jacobus van Wouwe
Mathew Varghese
Atheendar Venkataramani
Hans Verhoef
Cecile Viboud
Pieter Visser
Arnold Von Eckardstein
Charles Vorhees
Bradley Wagenaar
Gebhard Wagener
Stefan Wagner
Martin Walker
Richard Walker
Chris Wallace
David Wallon
Claire Wang
Wei Wang
James Wason
Graham Watt
Rob N.M. Weijers
Peter Weina
Steven Weisbord
Sheri Weiser
Kathleen Welsh-Bohmer
Nomi Werbeloff
Perla Werner
Lawrence Whalley
Peter White
Max Wicha
Nicholas Wilcken
Katherine Wild
Sarah Wild
Joshua Willey
Peter Williams
Lindsay Wilson
Peter Wilson
Monte Winslow
Daniel Witte
Bruce Wolfe
Charles Wolfe
Frank Wolters
Charles Woodrow
Alexander Wree
Caroline Wright
Joe Wu

Hannah Wunsch
Ming Xian
Zuoshang Xu
Jingwen Yan
Tse-Chuan Yang
Laura Yates
Sara Yeatman
Sze Lin Yoong
Yanglu Zhao
Hao Zhu
Huachen Zhu
Cathy Zimmerman
Deborah Zion
Laurance Zitvogel
Susan Zolla-Pazner
Alan Zonderman
